# Muscle Mitochondrial Uncoupling Dismantles Neuromuscular Junction and Triggers Distal Degeneration of Motor Neurons

**DOI:** 10.1371/journal.pone.0005390

**Published:** 2009-04-30

**Authors:** Luc Dupuis, Jose-Luis Gonzalez de Aguilar, Andoni Echaniz-Laguna, Judith Eschbach, Frédérique Rene, Hugues Oudart, Benoit Halter, Caroline Huze, Laurent Schaeffer, Frédéric Bouillaud, Jean-Philippe Loeffler

**Affiliations:** 1 Inserm, U692, Laboratoire de Signalisations Moléculaires et Neurodégénérescence, Strasbourg, France; 2 Université de Strasbourg, Faculté de Médecine, UMRS692, Strasbourg, France; 3 Hôpitaux Universitaires de Strasbourg, France; 4 DEPE, IPHC, Strasbourg, France; 5 Equipe Différenciation Neuromusculaire, IFR128, UMR5161, ENS Lyon, CNRS, INRA, Université de Lyon, Lyon, France; 6 BIOTRAM, Université Paris Descartes, CNRS UPR9078, Faculté de Médecine Necker-Enfants Malades, Paris, France; Tufts University, United States of America

## Abstract

**Background:**

Amyotrophic lateral sclerosis (ALS), the most frequent adult onset motor neuron disease, is associated with hypermetabolism linked to defects in muscle mitochondrial energy metabolism such as ATP depletion and increased oxygen consumption. It remains unknown whether muscle abnormalities in energy metabolism are causally involved in the destruction of neuromuscular junction (NMJ) and subsequent motor neuron degeneration during ALS.

**Methodology/Principal Findings:**

We studied transgenic mice with muscular overexpression of uncoupling protein 1 (UCP1), a potent mitochondrial uncoupler, as a model of muscle restricted hypermetabolism. These animals displayed age-dependent deterioration of the NMJ that correlated with progressive signs of denervation and a mild late-onset motor neuron pathology. NMJ regeneration and functional recovery were profoundly delayed following injury of the sciatic nerve and muscle mitochondrial uncoupling exacerbated the pathology of an ALS animal model.

**Conclusions/Significance:**

These findings provide the proof of principle that a muscle restricted mitochondrial defect is sufficient to generate motor neuron degeneration and suggest that therapeutic strategies targeted at muscle metabolism might prove useful for motor neuron diseases.

## Introduction

Amyotrophic lateral sclerosis (ALS) is the most frequent form of motor neuron disease in the elderly. Although most ALS cases occur sporadically, without known family history, a subset of these are genetically inherited. In particular, twenty percent of familial ALS cases are linked to mutations in the gene encoding the antioxidant enzyme Cu/Zn superoxide dismutase (SOD1). Transgenic mice expressing mutant forms of SOD1 (mSOD1 mice) recapitulate the phenotype of ALS and provide the only currently available faithful model of ALS [1], [2].

The mechanisms underlying the selective motor neuron degeneration in ALS remain elusive. Recent work in mSOD1 mice showed that motor neuron death is not cell autonomous and involves defects in other cell types than neurons [3], [4]. Consistent with this notion, recent evidence showed that the pathophysiology of ALS includes widely systemic defects in both patients and animal models. In particular, energy homeostasis is strikingly abnormal in ALS patients since both sporadic and familial ALS patients present with increased energy expenditure (hypermetabolism) and hyperlipidemia [5]–[8]. These systemic defects are in some yet unknown way linked to the disease process since hyperlipidemia correlates with increased survival in ALS patients [7]. Our work in animal models showed that mSOD1 mice were in energy deficit, with decreased adipose stores [9], [10]. This condition was elicited by increased energy expenditure, ie hypermetabolism, and was due to an increased consumption of both glucid and lipid nutrients in skeletal muscle [9], [10]. Early reduced muscle ATP levels, long before the onset of motor impairment further indicates impaired fuel processing [11]. Feeding these animals with a fat enriched diet replenished, to some extent, adipose stores and, most importantly, increased the lifespan and delayed muscle denervation of transgenic mSOD1 mice [9], [12]. Thus, ALS is associated with alterations of energy homeostasis which interferes with the pathological process.

Whether muscle hypermetabolism is sufficient *per se* to drive motor neuron degeneration remains however unknown. To address this question, we used here transgenic mice with muscular overexpression of uncoupling protein 1 (UCP1), a mitochondrial protein typically present in brown adipose tissue that induces non-shivering thermogenesis by uncoupling mitochondrial electron transport from ATP synthesis [13], [14], [15]. These animals naturally displayed an age-dependent deterioration of both the presynaptic and postsynaptic compartments of the neuromuscular junction (NMJ), and progressive signs of denervation that eventually led to mild late-onset motor neuron pathology in the spinal cord. NMJ regeneration and functional recovery were profoundly delayed following injury to the sciatic nerve and mutant SOD1 pathology was exacerbated by muscle UCP1 expression. These findings show that a relatively mild mitochondrial dysfunction in muscle recapitulates the earliest pathological events in ALS.

## Results

### Mitochondrial uncoupling leads to muscle weakness but preserves muscle structure

**Figure 1:**
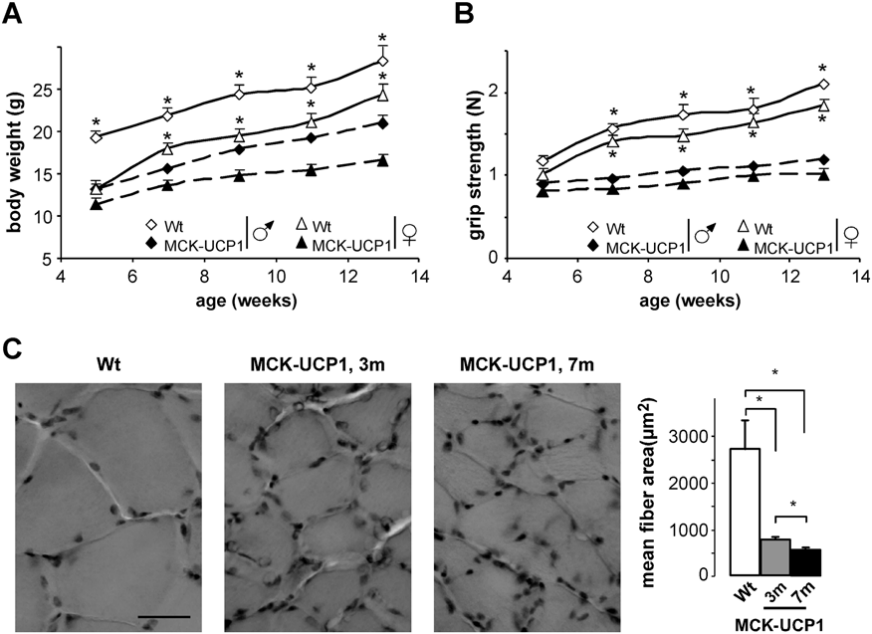
Muscle phenotype of MCK-UCP1 mice. A: body weight of male (empty diamonds) and female (empty triangles) wild-type mice, and male (filled diamonds) and female (filled triangles) MCK-UCP1 mice as a function of time. *, p<0.05 vs corresponding MCK-UCP1 (n = 7 mice per group). B: grip strength of mice as in A. *, p<0.05 vs corresponding MCK-UCP1 C: representative photomicrographs showing hematoxylin and eosin staining of gastrocnemius from wild-type mice (Wt) or MCK-UCP1 mice at 3 months (3 m) and 7 months (7 m) of age. The right panel shows the quantification of mean fiber area. *, p<0.05 (n = 4 mice per group).

**Figure 2:**
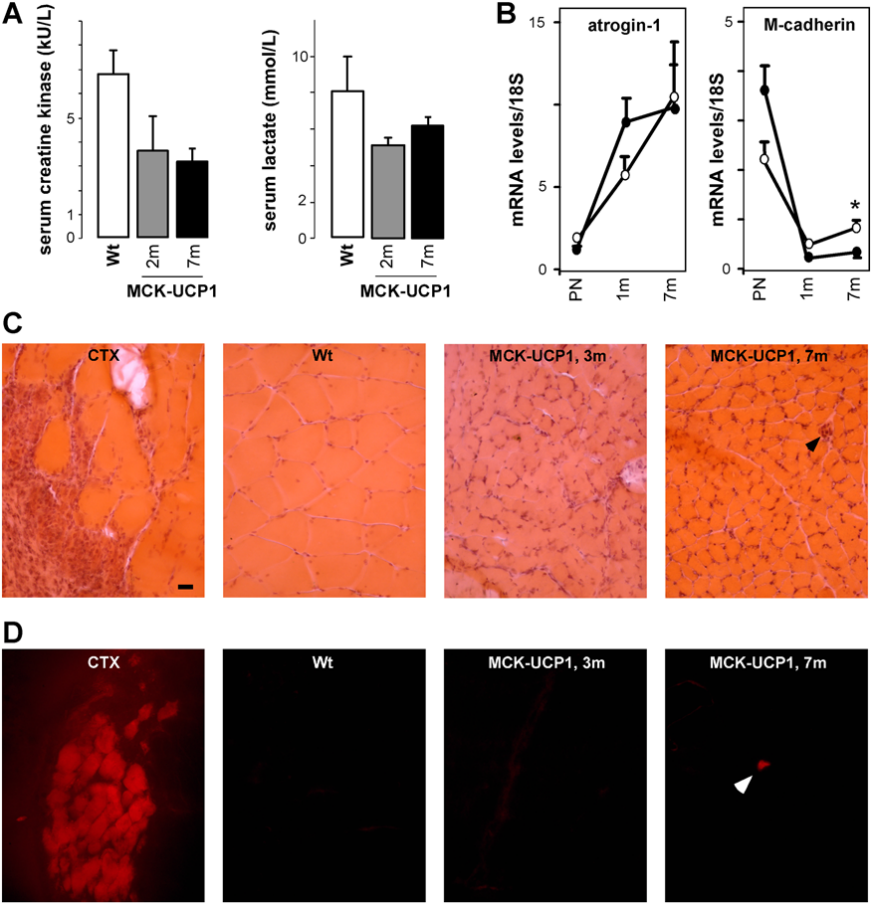
MCK-UCP1 do not display muscle dystrophy. A: serum creatine kinase (left panel) and lactate (right panel) concentrations in wild-type (Wt) or MCK-UCP1 mice at 2 months (2 m) and 7 months (7 m) of age. Non-significant differences (n = 8 mice per group). B: mRNA levels of atrogin-1 and M-cadherin in gastrocnemius from wild-type (empty circles) or MCK-UCP1 (black circles) mice at perinatal (PN), 1 month (1 m) and 7 months (7 m) of age. *, p<0.05 vs corresponding wild-type (n = 3–8 mice per group). C: representative photomicrographs showing hematoxylin and eosin staining of gastrocnemius from cardiotoxin-injured (48 hours after injury, CTX), wild-type (Wt) or MCK-UCP1 mice at 3 months (3 m) and 7 months (7 m) of age. Three mice per group were analyzed. A necrotic myofiber is indicated by an arrow. D: Evans blue dye staining as visualised by fluorescence in gastrocnemius from cardiotoxin-injured (48 hours after injury, CTX), wild-type (Wt) and MCK-UCP1 mice at 3 months (3 m) and 7 months (7 m) of age. Three mice per group were analyzed. Only a few necrotic myofibers could be detected in 7-month-old MCK-UCP1 mice (arrow).

To achieve a chronic muscle-restricted uncoupling, we used mice overexpressing UCP1 under the control of the promoter of the muscle creatine kinase (MCK) gene. Previous work on these MCK-UCP1 mice had shown slight decreases in both mitochondrial membrane potential (from 170 mV to 160 mV) and respiratory control ratio (from 3.3 to 2.5), which resulted in a mild phenotype primarily characterized by diminished muscle mass and fast-to-slow muscle fiber type switching in muscles such as the gastrocnemius [14]. As a first step toward the characterization of the neuromuscular phenotype in these animals, we sought to replicate and extend these results. Consistent with this, we found that MCK-UCP1 mice were lighter (figure 1A), and the cross-sectional surface of gastrocnemius myofibers showed a dramatic age-dependent reduction (figure 1C), which could account for their decreased grip strength (figure 1B). Since Han and collaborators [16] had previously reported that mice overexpressing high levels of UCP1 suffer from a prominent myopathic disorder, we checked whether the reduction in muscle fiber size observed in our animals could reflect a degenerative process. Serum concentrations of creatine kinase and lactate, an increase of which is indicative of muscular dystrophy [17], were not increased in MCK-UCP1 mice but rather tended to decrease, as compared to wild-type littermates (figure 2A). In addition, we did not detect the presence of myofibers containing centrally located nuclei (data not shown), that would be a sign of the regenerative process observed in muscular dystrophy [17]. In agreement with these findings, MCK-UCP1 muscles did not upregulate the satellite cell marker M-Cadherin, which is typically found in such regenerative processes (figure 2B). Necrotic myofibers, another hallmark of muscular dystrophy [17], can be easily identified by their permeability to Evans blue dye. In control experiments, a huge amount of Evans blue positive myofibers were observed in wild-type muscles following cardiotoxin injury, but only very few atrophic myofibers (less than 1 per field) were detected in 7-month-old MCK-UCP1 mice (figure 2D). Finally, mRNA levels of atrogin-1, an ubiquitin-ligase whose expression is strongly upregulated upon muscle wasting [18], appeared unchanged in MCK-UCP1 mice, as compared to wild-type littermates (figure 2B). Altogether, these findings confirm that mild mitochondrial uncoupling in MCK-UCP1 mice does not cause a dystrophic or massive proteolytic degeneration in skeletal muscle.

### Muscle mitochondrial uncoupling affects the integrity of AchR clustering

**Figure 3:**
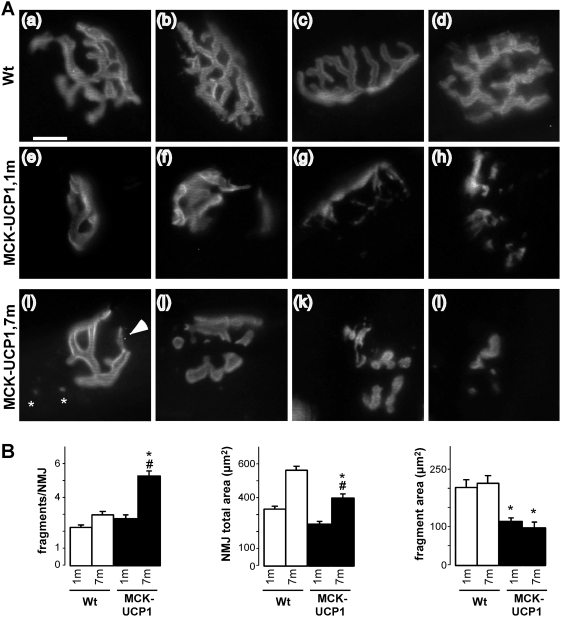
Aging MCK-UCP1 mice show progressive NMJ deterioration. A: representative photomicrographs showing NMJ morphology in gastrocnemius from wild-type (Wt) or MCK-UCP1 mice. Note the pretzel-shaped morphology of wild-type NMJs either at 1 month (a, b) or 7 months of age (c, d). In 1-month-old (1 m) MCK-UCP1 mice, NMJs appeared smaller and, in some rare cases, fragmented (e-h). At 7 months of age (7 m), thinner cisternae (arrow in i), ectopic AchR clusters (star in i), fragmented NMJs (j,k) and degenerated postsynaptic apparatus (l) were observed. Scale bar, 20 µm. B: quantitative morphometry of NMJs in gastrocnemius from wild-type (empty columns) or MCK-UCP1 (black columns) mice at 1 month (1 m) and 7 months (7 m) of age. *, p<0.05 vs corresponding wild type (n = 5 mice per group).

We next studied in detail the morphology of the NMJ in both 1- and 7-month-old mice. In wild-type muscle fibers, AchR clusters were organized in a classical pretzel-shaped structure, either at 1 (figure 3A, picture a and b) or 7 months of age (figure 3A, picture c and d). In contrast, the postsynaptic primary gutters in MCK-UCP1 fibers appeared less ramified already at 1 month of age (figure 3A, pictures e-h), and, in very rare cases, fragmented (figure 3A, picture h). At 7 months of age, fragmentation was very frequent, and the size of the postsynaptic apparatus had decreased, as compared to wild-type mice (figure 3A, pictures i–l). Moreover, in the few NMJs presenting with normal primary gutters at this age, thinner cisternae (arrowhead in figure 3A, picture i) and neighboring ectopic AchR clusters (asterisks in figure 3A, picture i) could be detected. Quantitative analysis of these phenomena corroborated the histological observations and revealed a significant deterioration of NMJ morphology in MCK-UCP1 mice (figure 3B). In all, these findings indicate that NMJ morphology progressively deteriorates with age upon chronic muscle mitochondrial uncoupling.

### Muscle mitochondrial uncoupling triggers abnormal muscle electrical activity

**Figure 4:**
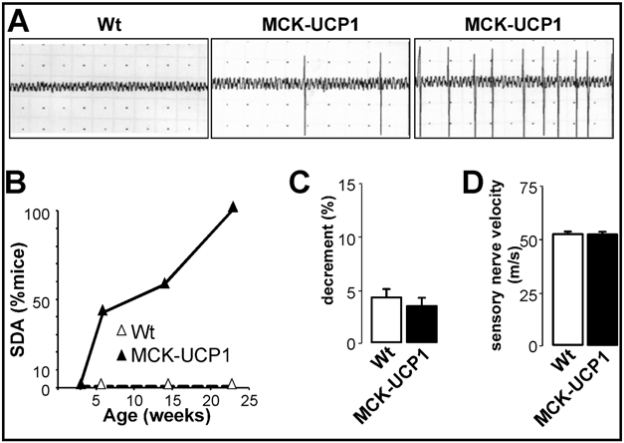
Electromyographic features of MCK-UCP1 mice. A: representative electromyography recordings in gastrocnemius from a 7-month-old wild-type mouse (left panel), and two 7-month-old MCK-UCP1 mice showing weak (middle panel) and intense (right panel) altered electrical activity. B: fraction of mice presenting with aberrant spontaneous denervation activity in at least one muscle territory. Wild-type mice (empty triangles), MCK-UCP1 mice (filled triangles) (n = 6–10 mice per group). C: decrement observed in a repetitive stimulation test in gastrocnemius from 7-month-old wild-type (Wt) and MCK-UCP1 mice (n = 6 mice per group). D: sensory nerve velocity in 7-month-old wild-type (Wt) and MCK-UCP1 mice (n = 6 mice per group).

Next we asked whether the morphological abnormalities of the postsynaptic apparatus in MCK-UCP1 mice could be secondary to muscle denervation. To this end, we performed longitudinal electromyography studies. Before 6 weeks of age, MCK-UCP1 mice showed normal electrical activity recordings, as compared to wild-type animals (figure 4A), suggesting that NMJs were functional and properly innervated at that age. Later on, however, a subset of mice started to exhibit fasciculations, which gradually progressed in older animals to strongly altered patterns characterized by fibrillations, fasciculations and myotonic discharges (figure 4A). After 7 months of age, all the mice were affected (figure 4B). To test whether this abnormal electrical activity was the reflect of altered neurotransmission at the NMJ, as occurs for example in congenital myasthenic syndromes, we recorded the muscle evoked response to repetitive nerve stimulation. If neuromuscular transmission was affected, a sharp decrease (>10%) in the amplitude of the electrical response should be observed after repeated stimulations, but no differences were detected between MCK-UCP1 and wild-type mice (figure 4C), indicating that neuromuscular transmission did not appear compromised. Additionally, we measured caudal sensory nerve velocity to determine whether sensory abnormalities could be observed, but found no changes in MCK-UCP1 mice (figure 4D).

### Muscle mitochondrial uncoupling triggers endplate denervation and distal axonal degeneration

**Figure 5:**
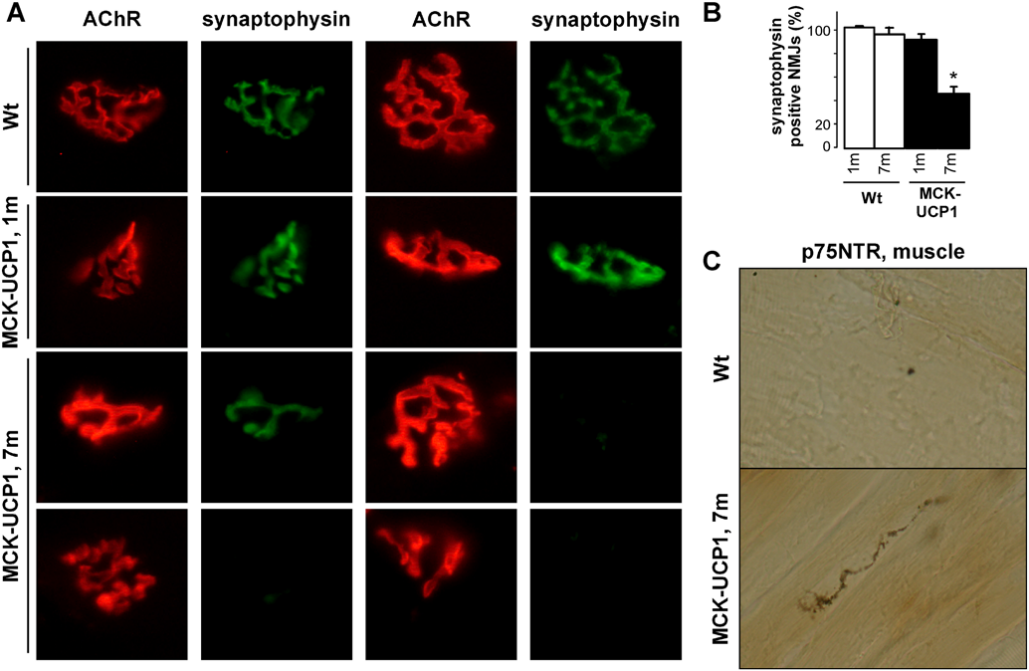
Aging MCK-UCP1 mice show progressive denervation and distal axonal degeneration. A: representative photomicrographs showing AchR (red) and synaptophysin (green) staining in gastrocnemius from wild-type (Wt) or MCK-UCP1 mice at 1 month (1 m) and 7 months (7 m) of age. Scale bar, 20 µm. B: quantification of the percentage of synaptophysin positive NMJs as shown in D, *, p<0.05 vs wild-type. (n = 4 mice per group). C: representative photomicrograph showing p75NTR immunostaining in gastrocnemius from 7-month-old wild-type (Wt) or MCK-UCP1 mice. Scale bar, 20 µm.

The presence of abnormal electromyography patterns together with the observed morphological changes in the postsynaptic apparatus from old MCK-UCP1 mice could reflect denervation. Immunohistochemical analysis of the pre- and postsynaptic compartments of the NMJ revealed complete overlapping of synaptophysin and alpha-bungarotoxin staining in 1-month-old MCK-UCP1 mice, whereas 60% of the alpha-bungarotoxin positive NMJs in 7-month-old MCK-UCP1 mice lacked synaptophysin labeling (figure 5A–B), thus suggesting loss of the presynaptic button. To further confirm these observations, muscle sections were stained with an antibody directed against p75NTR, which is typically overexpressed in Schwann cells surrounding degenerating motor endings [19]. p75NTR positive nerve fibers were systematically detected in old, but not young, MCK-UCP1 mice (figure 5C). Altogether, these findings show that MCK-UCP1 mice are afflicted by a distal, age-related axonopathy.

### Muscle mitochondrial uncoupling triggers spinal cord astrocytosis and mild motor neuron degeneration

**Figure 6:**
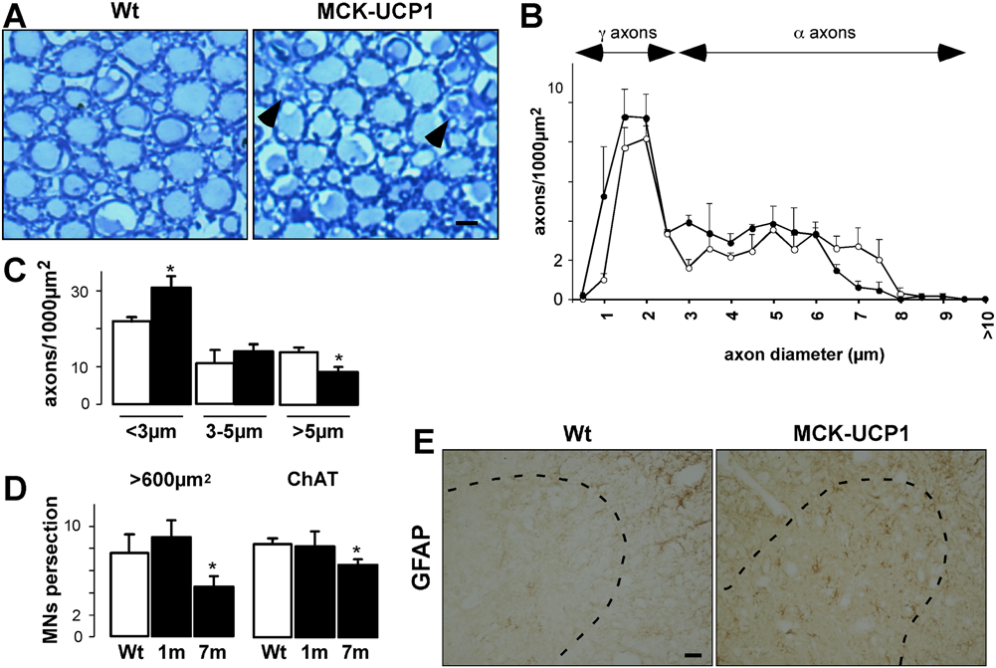
Mild late onset motor neuron degeneration in MCK-UCP1 mice. A: representative photomicrographs showing ventral roots from wild-type (Wt) and MCK-UCP1 mice. Note the presence of two degenerating axons in the MCK-UCP1 picture (arrows). B: distribution of the calibers of axons in the ventral roots from wild-type (empty circles) or MCK-UCP1 (filled circles) mice. Small-caliber axons represent gamma-axons, which innervate muscle spindles, whereas large-caliber axons represent alpha-axons, which innervate skeletal muscles. Note the decrease in the population of the largest caliber axons in MCK-UCP1 ventral roots. C: size distribution of axons in the ventral roots from 7-month-old wild-type (empty columns) and MCK-UCP1 (filled columns) mice. *, p<0.05 vs wild-type (n = 3 mice per genotype). D: quantification of the number of motor neurons in the lumbar spinal cord form wild-type (Wt) and MCK-UCP1 (MCK-UCP1) mice at 3 months (3 m) and 7 months (7 m) of age, after toluidine blue-staining (left panel) and ChAT immunoreactivity (right panel). *, p<0.05 vs wild-type (n = 5 mice per group). E: representative photomicrographs showing GFAP immunoreactivity in the lumbar spinal cord from 7-month-old wild-type (Wt) or MCK-UCP1 mice. Ventral horns are indicated by dashed lines. Scale bar, 20 µm.

The existence of a NMJ pathology in aging MCK-UCP1 mice prompted us to determine whether any abnormalities could be detected in the ventral root axons and their corresponding spinal cord motor neuronal cell bodies. Ventral roots from MCK-UCP1 mice appeared grossly normal, but showed sparse axonal degeneration (figure 6A). A more detailed analysis of the distribution of axonal calibers revealed an almost 50% reduction in the amount of large (>5 micrometers in diameter) caliber axons, together with a concomitant increase in the amount of very small caliber, presumably degenerating axons (figure 6B–C). Motor neuron counting was performed in the ventral horns of the lumbar spinal cord at 3 and 7 months of age, by means of a morphometric approach based on the cross-sectional area of the cells (figure 6D, left) or by direct identification of choline acetyltransferase positive motor neurons (figure 6D, right). Both methods demonstrated a significant 20–30% decrease in the number of motor neurons in 7-month-old MCK-UCP1 mice, as compared to 3-month-old MCK-UCP1 or wild-type animals. The presence of astrocytosis in the lumbar spinal cord, which is typically associated with neuronal suffering [20], was assessed using an antibody directed against the astrocyte marker GFAP. An increase of GFAP positive astrocytes was observed in 7-month-old MCK-UCP1 mice, as compared to wild-type littermates (figure 6E). In all, these findings strongly suggest that chronic muscle-restricted mitochondrial uncoupling is able to induce a mild but clear motor neuron pathology, likely as a result of the progressive degenerative process initiated at the NMJ.

### Muscle mitochondrial uncoupling strongly increases agrin-induced AchR clustering

**Figure 7:**
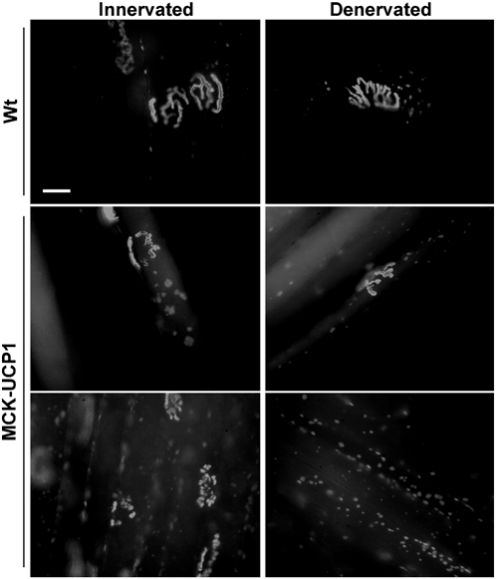
Old MCK-UCP1 mice show increased AchR clustering in response to agrin. representative photomicrographs showing AchR clusters in contralateral (innervated) and ipsilateral (denervated) tibialis anterior from wild-type (Wt) or MCK-UCP1 mice 8 days after sciatic nerve axotomy and 15 days after recombinant agrin injection. Scale bar, 20 µm.

The first pathological event observed in MCK-UCP1 mice appeared to be the dismantlement of the post-synaptic apparatus. Since acetylcholine receptor (AchR) clustering is likely to be an energy-dependent process, we hypothesized that mitochondrial uncoupling occuring in MCK-UCP1 muscles lead to an inability of these muscles to maintain AchR clusters with age. To test this hypothesis, we determined whether old MCK-UCP1 mice were still able to newly form ectopic AchR clusters. Neural agrin, via activation of muscle specific kinase (MuSK), is responsible for NMJ formation and stability [21], and is known to trigger ectopic AchR clustering upon intramuscular injection, the response being further enhanced by muscle denervation. In both innervated and denervated muscle, intramuscular injection of agrin stimulated ectopic AchR clustering much more efficiently in MCK-UCP1 mice than in wild-type animals, the largest clusters being observed in innervated MCK-UCP1 muscle (figure 7). In particular, large AchR clusters were frequently detected in the extra junctional area in MCK-UCP1 muscle fibers, whereas only small AchR clusters could be observed and were closely confined to the synaptic region in wild-type fibers. These findings show that, contrary to our initial hypothesis, aged MCK-UCP1 mice are still able to efficiently respond to NMJ-inducing stimulatory nerve factors and suggest that the NMJ defects of these mice are rather presynaptic.

### Muscle mitochondrial uncoupling delays motor recovery after lesion

**Figure 8:**
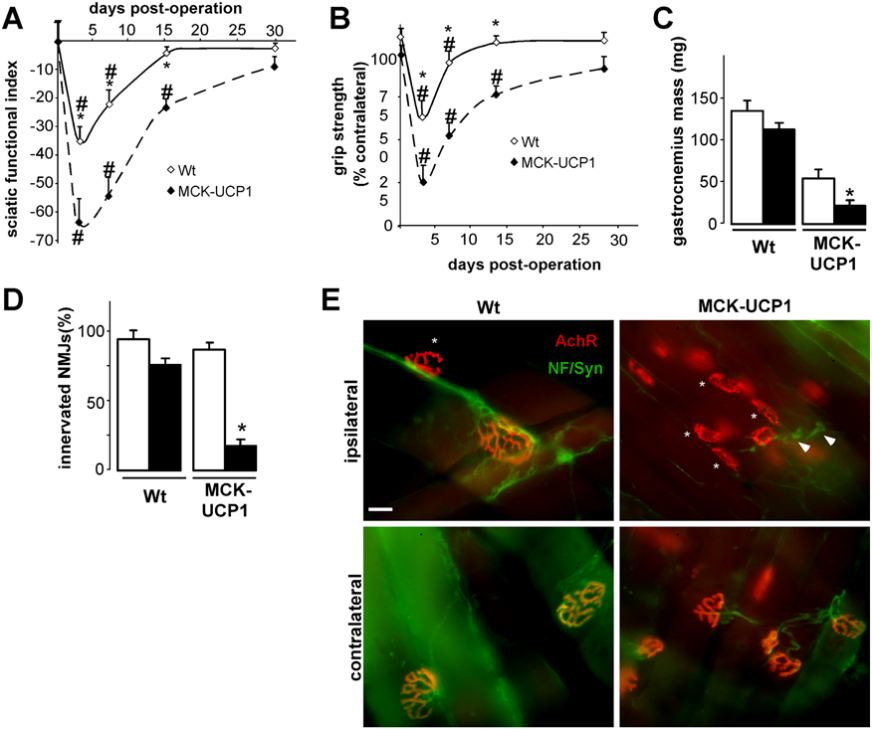
MCK-UCP1 mice show delayed functional recovery after nerve crush. A–B: Sciatic functional index (A) and grip strength (B) in 1-month-old wild-type (empty diamonds) or MCK-UCP1 mice (filled diamonds) after sciatic nerve crush, performed at day 0. Grip strength was expressed as the percentage of ipsilateral strength relative to the contralateral limb. *, p<0.05 vs corresponding wild type; #, p<0.05 vs day 0 (n = 12 mice per group). C: Mass of contralateral (empty columns) and ipsilateral (black columns) gastrocnemius from wild-type (Wt) or MCK-UCP1 mice 30 days after sciatic nerve crush. *, p<0.05 vs contralateral muscle (n = 5 mice per group). D: quantification of the percentage of synaptophysin positive NMJs as shown in E, *, p<0.05 vs wild-type. (n = 5 mice per group). E: representative photomicrographs showing AchR (red) and neurofilament/synaptophysin (green) staining in contralateral and ipsilateral gastrocnemius from wild-type (Wt) or MCK-UCP1 mice 8 days after sciatic nerve crush (e) and quantification of the experiment (d). Note the denervated endplates (stars) and the retracted nerve fibers (arrowheads). Scale bar, 20 µm.

Since AchR clustering is efficiently maintained in MCK-UCP1 mice, we assessed whether axonal regeneration upon injury was normal. To this aim, we performed sciatic nerve crush studies. This experimental model is typically characterized by axonal degeneration and subsequent reinnervation, generally completed within 2 weeks post-lesion. After nerve crush, motor recovery was evaluated by the sciatic functional index (SFI), a scale based on the shape of footprints, and grip strength [22]. In wild-type mice, the SFI decreased to a minimum 3 days post-operation and then progressively recovered to reach a normal value at day 15. In MCK-UCP1 mice, the decrease of the SFI was much more pronounced, and recovery was greatly delayed and hardly completed 30 days after lesion (figure 8A). Consistent with this, grip strength of the operated limb showed a quite similar evolution (figure 8B). In addition, the accompanying recovery of muscle mass, which was complete 1 month post-operation in wild-type mice, appeared significantly impaired in MCK-UCP1 animals (figure 8C). To determine whether the retardation in functional recovery was due to delayed reinnervation, we performed double neurofilament/synaptophysin immunostaining along with alpha-bungarotoxin labeling to examine the aspect of the NMJs after nerve crush. NMJs from operated wild-type mice had almost completely recovered 8 days after lesion, whereas most NMJs in MCK-UCP1 mice remained denervated and appeared smaller (figure 8D–E). Altogether, these findings show that mitochondrial uncoupling in skeletal muscle limits considerably axonal regeneration after lesion.

### Muscle mitochondrial uncoupling exacerbates motor neuron disease in mice

**Figure 9:**
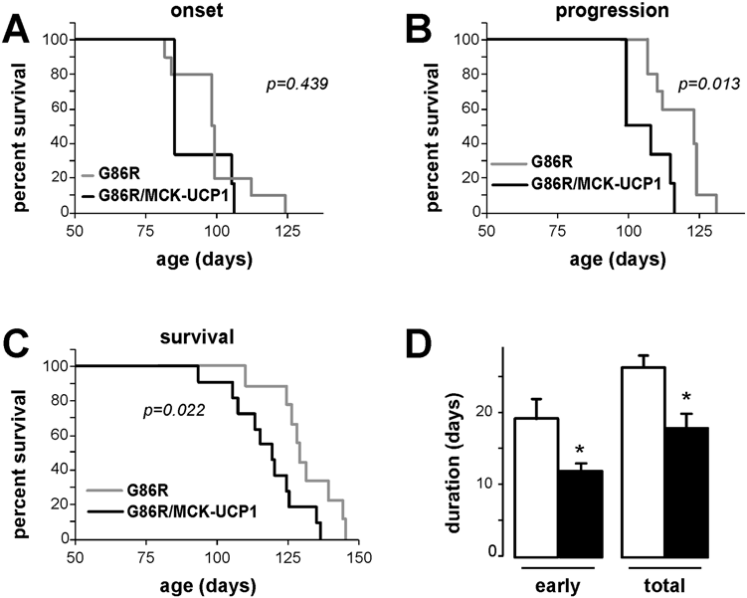
UCP1 overexpression exacerbates motor neuron disease in mice. A–C: Kaplan-Meier curves showing the cumulative probability of disease onset (age at the peak of body mass prior to decline, A), progression through early disease stage (when maximal body mass decreases by 10%, B), and total survival (C). The p-values, as calculated using the logrank test, are shown. D: duration of early disease stage (left) and total duration of disease (right) in SOD1(G86R) mice (empty columns) and compound SOD1(G86R)/MCK-UCP1 mice (filled columns). *, p<0.05 vs corresponding SOD1(G86R).

To further explore the negative influence of muscle mitochondrial uncoupling on motor neuron degeneration, we analysed whether muscle UCP1 overexpression could change the pattern of disease in a transgenic mouse model of amyotrophic lateral sclerosis (ALS). To this end, we crossbred MCK-UCP1 mice with SOD1(G86R) mice, a transgenic line harboring an ALS-linked mutated form of Cu/Zn-superoxide dismutase [23], [24]. To monitor disease progression, we followed the characteristic decline in peak body mass observed in these animals as the earliest symptom that coincides with initial axonal retraction from NMJs. Then, we defined an early stage of disease as the time from onset until maximals body mass has decreased by 10% ([3], [9] and our own observations on the SOD1(G86R) mouse line). Based on these criteria, no differences were detected in disease onset (figure 9A). However, both progression from onset through early disease (figure 9B) and total survival (figure 9C) were significantly shortened in heterozygous SOD1(G86R)/MCK-UCP1 mice, as compared to SOD1(G86R) mice (figure 9D). Altogether, these data indicate that muscle-specific mitochondrial uncoupling is, in itself, sufficient to deteriorate NMJ stability and hence contribute to the progression of motor neuron disease.

## Discussion

This study demonstrates that a mild uncoupling of mitochondrial respiration in skeletal muscle profoundly affects NMJ stability and function and leads to distal degeneration of motor neurons. These findings show that muscle mitochondria are crucially involved in NMJ maintenance and are potentially relevant to the pathophysiology of MNDs.

### Muscle uncoupling leads to distal axonopathy and late onset motor neuron degeneration

Mild muscle-restricted mitochondrial uncoupling had little effect on muscle overall structure, despite reduced mass and mild mitochondrial myopathy (Han and our data not shown). In young mice, mitochondrial uncoupling did not appear to interfere with the development of a normal, pretzel-shaped innervated NMJ. However, as shown here by using a combination of histological and electrophysiological techniques, these NMJs progressively underwent denervation upon aging. This in turn leads to a distal axonopathy that progressed with age to a limited but clear motor neuron degeneration. Our results indicate that the progressive deterioration of NMJs in aging MCK-UCP1 mice results from an intrinsic instability of the axon rather than from a post-synaptic dismantlement of the NMJ. Two lines of results are consistent with this notion. First, the formation of AchR clusters in response to neural agrin was not impaired but rather enhanced in MCK-UCP1 muscles (figure 7), suggesting that the complex postsynaptic muscular machinery responsible for AchR clustering is not impaired. Second, functional recovery after nerve injury was considerably delayed in MCK-UCP1 muscles (figure 8), suggesting a rather inefficient nerve terminal regeneration and a severe impairment of the presynaptic mechanisms that underlie axonal regrowth. A very plausible explanation for the progressive denervation and impaired reinnervation observed in MCK-UCP1 mice is that mitochondrial uncoupling in muscle modifies the production of retrograde signals required for presynaptic motor ending stability. Since, at present, little is known on the nature of the signals produced by an adult muscle fiber to maintain the stability of the neuromuscular contact, the precise signalling mechanisms elicited by mitochondrial uncoupling and their relative contribution to the pathology of these mice will require further investigations. Such studies will undoubtly shed light on new retrograde muscle to nerve survival signalling pathways. It is important to note that our current results are compatible with two pathological scenarios: first, the NMJ alterations might be due to cell autonomous effects of transgene expression. In this scenario, mitochondrial uncoupling in a given muscle fiber would locally lead to the degeneration of its innervating axon. Alternatively, since transgene expression in muscles leads to broadly systemic effects, these systemic alterations could weaken NMJs and neuromuscular contacts. Indeed, caloric restriction is able to accelerate the outcome in mSOD1 mice [25], suggesting that interventions ameliorating systemic energy metabolism might also weaken motor neurons.

### Muscle mitochondrial uncoupling does not lead to paralysis or premature death

Although displaying a massive destruction of the NMJs, MCK-UCP1 mice do not display a full blown fatal motor neuron disease. This apparently paradoxical situation may rest on the fact that not all the muscles in MCK-UCP1 mice are affected by UCP1 overexpression. In fact, numerous muscles beds, including the soleus, the diaphragm and heart muscles appeared relatively normal in these animals, despite that they express almost equivalent levels of UCP1 protein than in severely affected muscles such as the gastrocnemius or the tibialis anterior muscles ([14] and data not shown). From their seminal observations, Couplan and collaborators concluded that muscles undergoing regular contractions, and therefore resting on a continuous mitochondrial ATP production, are protected from the uncoupling activity of UCP1 [14]. This is consistent with the idea that regular contractions trigger a sustained low mitochondrial potential in these muscles that inhibits the protonophoric activity of UCP1. Thus, despite being overexpressed in all muscles, the transgene only affects fast-twitch fibers, which are predominant in the gastrocnemius [13], [14]. This tissue-restricted pathology is likely to explain why the phenotype described here is compatible with a normal lifespan and has not been reported before.

### Does skeletal muscle contribute to motor neuron degeneration in ALS?

Our data suggest that muscle selective alterations in mitochondrial function might initiate NMJ pathology and participate in triggering motor neuron degeneration in ALS. The pathology of MCK-UCP1 mice begins with NMJ destruction and distal axonopathy and then progresses towards astrocytosis in the spinal cord and mild motor neuron loss, both typical features of motor neuron diseases. Importantly, in ALS too, the destruction of the NMJ appears as the first detectable pathophysiological event and precedes axonal degeneration and motor neuron death in mutant SOD1 mice [26]. In MCK-UCP1 mice, NMJ destruction is due to a muscle specific alteration in mitochondrial function and similar alterations have been documented in the muscle of both sporadic ALS patients and mutant SOD1 mice [27]. Mitochondrial dysfunction in muscle might be either directly caused by mutant SOD1 expression in muscle or indirectly through mutant SOD1 expression in other cell types. Consistent with the first hypothesis, a recent study by Dobrowolny and collaborators showed that restricted mutant SOD1 expression in muscle is sufficient to trigger muscle atrophy and mitochondrial dysfunction. Similar to the situation in MCK-UCP1 mice, muscle restricted expression of mutant SOD1 triggered some of the pathological aspects of ALS in the spinal cord, including oxidative stress and gene expression profiles consistent with microgliosis [28]. In view of the parallel phenotypes between mutant SOD1 and MCK-UCP1 mice, one can suggest that mutant SOD1 expression in skeletal muscle is able to trigger axonal degeneration through the direct implication of mitochondria. This hypothesis is however not completely consistent with the available evidence. Two studies showed that a loss of mutant SOD1 expression in muscle did not change disease outcome in mutant SOD1 mice [29], [30], suggesting that either minute quantities of remaining mSOD1 in muscle are still sufficient to trigger the disease or that the knockdown of mutant SOD1 in muscle was performed too late in the course of the pathology. Whatever the exact contribution of muscle mSOD1 to mitochondrial dysfunction in these mice, the elucidation of the mechanisms underlying motor neuron pathology in MCK-UCP1 mice shed light on the early pathological events of ALS and might then open avenues for potential therapeutic strategies in ALS and other motor neuron disorders.

## Materials and Methods

### Animals and surgery

Heterozygous MCK-UCP1 mice were bred on the original genetic background and identified by tail DNA genotyping as previously described [14]. Transgenic mice expressing the murine G86R SOD1 mutation were obtained in our animal facility and genotyped as described [9]. Disease monitoring was performed according to previous reports [3], [9]. Wild-type littermates served as controls. Animals were maintained on a 12 h light/12 h dark cycle, and received food and water *ad libitum*. For histological analysis of spinal cord tissue, animals were deeply anesthesized with 1 mg/kg body weight ketamine chlorhydrate and 0.5 mg/kg body weight xylazine, and transcardially perfused with 4% paraformaldehyde in 0.1 M phosphate buffer pH 7.4. Tissues were then quickly dissected, post-fixed in 4% paraformaldehyde for 24 hours, and cryoprotected with 30% sucrose in PBS for 48 hours before cryostat sectioning. For histological analysis of muscle tissue, samples were fresh dissected and frozen in melting isopentane in dry ice. For biochemical analysis, animals were sacrificed and tissues were quickly dissected, snap frozen in liquid nitrogen and stored at −80°C until use. Sciatic nerve crush and axotomy were performed as described [31]. Briefly, mice were anesthesized as mentioned above, and hind limb skin was carefully shaved and incised at the mid thigh level. Sciatic nerve was exposed and either cut or crushed during 30 seconds. Skin was sutured, and animals were allowed to recover. Animal manipulation followed current EU regulations and was performed under the supervision of authorized investigators.

### Analysis of motor function

Grip strength measurements were performed using a Bioseb gripmeter following the manufacturer's instructions. Data were the mean of three successive trials per mouse. Grip strength was measured on the four limbs or on each individual hind limb for evaluation of functional recovery following sciatic nerve crush. The sciatic functional index (SFI) was calculated using the footprints of the operated mice as described by Schiaveto de Souza and collaborators [22].

### Biochemical measurements

Plasma was collected on heparinized capillaries, and lactate and creatine kinase levels were measured following standard clinical protocols.

### Skeletal muscle histology

Cryostat cross-sections 20 µm thick of gastrocnemius muscle were stained with hematoxylin and eosin using a standard protocol. For detection of necrotic myofibers, mice were intraperitoneally injected with 1% Evans Blue dye in saline as described [32]. Briefly, 16 hours after injection, mice were sacrificed, and muscles were immediately frozen in melting isopentane and cut in a cryostat. Sections were dipped into cold acetone, dried and mounted in non-fading medium until observation.

### Analysis of neuromuscular junction morphology

Unless otherwise indicated, muscle bundles were prepared under a binocular microscope, and labeled with rhodamine-conjugated alpha-bungarotoxin (Sigma, Saint-Quentin Fallavier, France) and Hoechst dye. When necessary, longitudinal sections of gastrocnemius muscle 40-µm thick were co-stained with rhodamine-conjugated alpha-bungarotoxin and antibodies directed against synaptophysin (Dako, Trappes, France), neurofilament (Sigma, Saint-Quentin Fallavier, France) or p75NTR (Chemicon International, Hampshire, UK), using a standard protocol as previously described [33]. Usually, 5–6 non-adjacent sections were examined per animal, which represented 100–150 analyzed NMJs per animal.

### Lower motor neuron counting

Motor neuron counts were obtained from the ventral horns of the lumbar spinal cord segments L3–L5, using at least 7 non-adjacent sections 10-µm thick per animal examined in a Nikon microscope at a 200× magnification [9]. Sections were stained with 1% toluidine blue in 5% sodium borate, and the cross-sectional areas of the stained cells were measured with the NIH Image software. Only those cells with an area ≥600 µm^2^ were considered as motor neurons. In a parallel set of sections, motor neurons were identified by immunostaining with an antibody directed against choline acetyltransferase (ChAT; Chemicon International). Counts of ChAT positive motor neurons were performed by an independent genotype-blinded observer.

### Electromyography

Electromyography recordings were made using a standard apparatus (Dantec, Les Ulis, France) as previously described [33]. Mice were anesthetized as mentioned above, and a monopolar needle electrode (diameter 0.3 mm; 9013R0312; Medtronic, Minneapolis, MN) was inserted into the tail of the mouse to ground the system. Recordings were made with a concentric needle electrode (diameter 0.3 mm; 9013S0011; Medtronic). Electrical activity was monitored on gastrocnemius muscles at both right and left sides for at least 2 min. Spontaneous activity was differentiated from voluntary activity by visual and auditory inspection. Voluntary activity is characterized by rather repetitive regular discharges, which disappear with relaxation of the muscle. Only spontaneous activity with a peak-to-peak amplitude of at least 50 µV was considered to be significant. The repetitive nerve stimulation test was performed to assess neuromuscular transmission. Supramaximal square pulses, of 0.2 ms duration, were delivered through a needle electrode to the sciatic nerve at the sciatic notch level. An anode needle was inserted at the base of the tail. The active recording needle electrode was inserted in the medial part of the gastrocnemius. Reference recording needle electrode was inserted over the Achilles tendon. The myoelectric signal was bandpass filtered (2 Hz–5 kHz) to eliminate artefacts. First, one set of 5, 3-Hz supramaximal stimulations was delivered continuously; CMAPs were recorded simultaneously for the same period. A decrease of more than 10% in the amplitude of the fifth evoked response compared to the initial (first) response was defined as a significant decremental response to a supramaximal stimulation. To elicit the sensory nerve action potential, the tail was strapped to a polystyrene board. The monopolar needle electrodes were inserted as follows: (a) the stimulating cathode was placed exactly two-thirds of the length of the tail, distally to its hairline, (b) the stimulating anode was inserted 3 mm distal to the stimulating cathode, (c) the recording cathode and anode were inserted 5 and 2 mm distal to the hairline of the tail, respectively, and (d) a ground electrode was inserted half way between the stimulating and the recording cathodes. Ten responses were averaged for each recording. The sensory NCV of the tail nerve was calculated from the latency of the stimulus artifact to the onset of the negative peak of the action potential elicited and the distance between the stimulating and the recording cathodes.

### Real time RT-PCR

Total RNA was extracted from gastrocnemius using Trizol (Invitrogen, Cergy-Pontoise, France) according to the manufacturer's instructions. cDNA synthesis was performed using 1 µg of total RNA (iScript cDNA Synthesis kit; Bio-Rad, Marne La Coquette, France). PCR analysis was carried out on a Bio-Rad iCycler System using iQSYBR Green Supermix. A specific standard curve was performed in parallel for each gene, and each sample was quantified in duplicate. PCR conditions were 3 min at 94°C, followed by 40 cycles of 45 s at 94°C and 10 s at 60°C. Data were analyzed using the iCycler software, and normalized to the 18S ribosomal subunit RNA.

### Recombinant agrin injections

Chicken neural mini-agrin [34] was cloned into the pCEP-Pu expression vector. The recombinant protein was targeted to the secretory pathway by the signal peptide of BM4O and it included a carboxy-terminal His6-tag. The carboxy-terminal included the 4- and 8-amino-acid inserts at the A/y and B/z sites and thus represents the neural isoform. The secreted recombinant agrin was purified on Ni-column (Ni-NTA agarose, Qiagen) from the culture medium of 293 EBNA cells stably transfected with the pCEP-Pu-agrin vector. Mice were anesthesized as mentioned above, and the tibialis anterior was bilaterally injected with 500 ng of recombinant agrin. Seven days after injection, mice were subjected to unilateral sciatic nerve axotomy and were sacrificed seven days after. Tibialis anterior muscles were immersed in 4% paraformaldehyde during 30 min and stored in PBS until use. Muscle bundles were prepared and stained for AchR as previously described.

### Statistical analysis

Data are expressed as the mean±SEM. Statistical analysis was accomplished using Student *t*-test or ANOVA followed by Tukey-Kramer multiple comparisons test. The logrank test was applied to compare Kaplan-Meier curves (PRISM version 4.0b; GraphPad, San Diego, CA). Differences at *P*<0.05 were considered significant.
